# Advances in the neurotoxicity of ecological pesticide maneb: mechanisms and implications for human health

**DOI:** 10.3389/fpubh.2026.1745443

**Published:** 2026-02-02

**Authors:** Yuping Xi, Zhibing Zhang, Ziting Lin, Kun Peng, Shuang Chen, Qihu Zhu, Yinglong Cao

**Affiliations:** 1 The College of Ethnology and Sociology, South-Central Minzu University, Wuhan, China; 2 Research Center for Environment and Health, Zhongnan University of Economics and Law, Wuhan, China; 3 School of Information Engineering, Zhongnan University of Economics and Law, Wuhan, China; 4 College of Life Science and Technology, Huazhong Agricultural University, Wuhan, China

**Keywords:** environmental residue, human exposure, maneb, neurotoxicity, Parkinson’s disease

## Abstract

Maneb, a typical dithiocarbamate pesticide, is widely used in the control of agricultural fungi in lands. Its residues with main metabolites in environmental media pose risks to environmental safety and human health. This research summarized the research progress on the environmental residues, human exposure levels, neurotoxic effects and mechanisms of maneb. Epidemiological studies have shown that exposure to maneb is associated with an increased risk of neurodegenerative diseases such as Parkinson’s disease (PD). *In vitro* and *in vivo* experiments have further demonstrated that exposure to maneb induces neurotoxic effects in cells and animals, involving multiple injury pathways and molecular mechanisms, including reduced cell viability, oxidative stress, mitochondrial dysfunction, synaptic dysfunction, and induction of apoptosis, ultimately leading to the degeneration of dopaminergic neurons. Future research can focus on the systemic comprehensive toxicity and interaction mechanisms of maneb to promote its safe application in agricultural production.

## 1 Introduction

**Figure 1:**
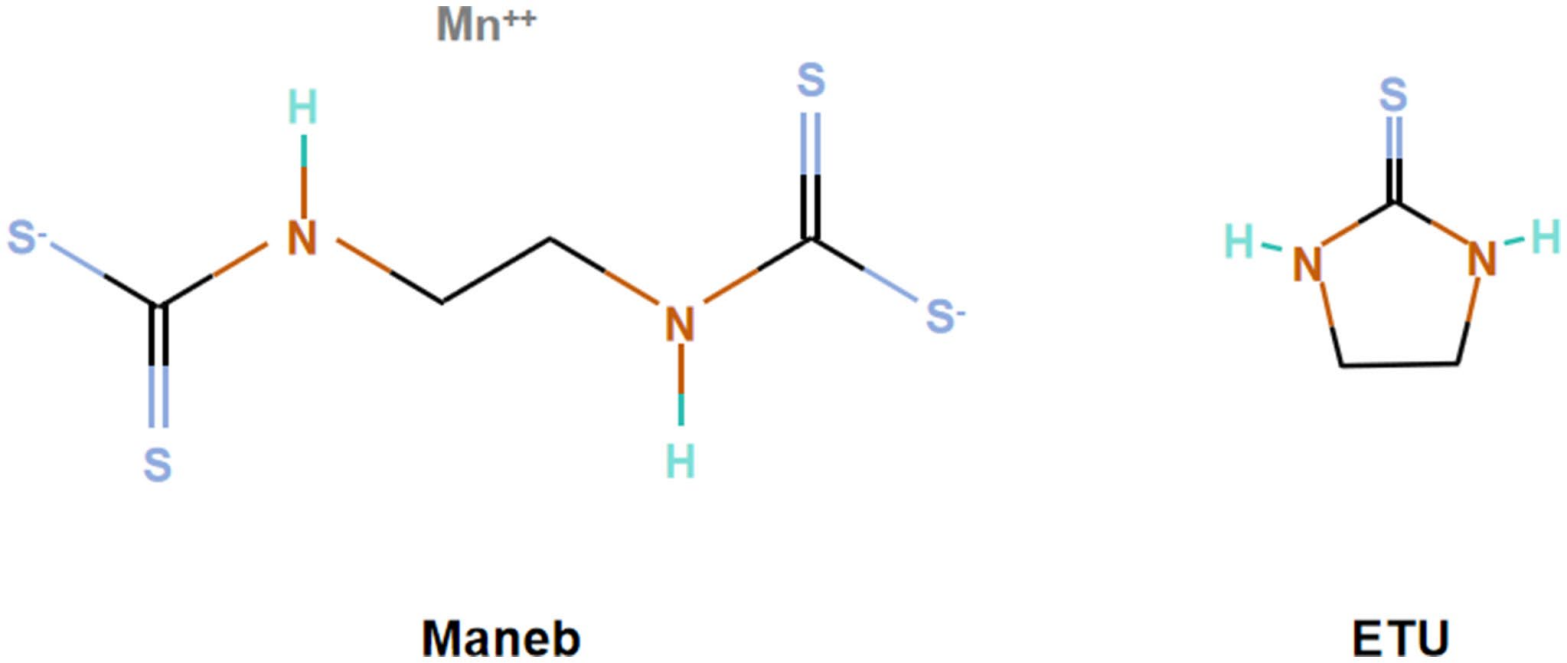
The molecular structure formula of maneb and ETU.

Manganese ethylene-bis-dithiocarbamate (maneb), a typical ethylene-bis-dithiocarbamate (EBDC) fungicide, was first registered as a broad-spectrum fungicide in the United States in 1962. Its molecular formula is C_4_H_6_N_2_S_4_·Mn, and it is a yellow crystalline powder that is insoluble in water and most organic solvents. It has a certain degree of photostability and decomposes after exposure to air or moisture for a period of time (1, 2). Maneb is composed of the organic ligand EBDC and free metal Mn^2+^. The main degradation product of its organic portion is ethylene thiourea (ETU), with the chemical formula C_3_H_6_N_2_S (3). The molecular structure formula of maneb and ETU are shown in Figure 1.

Maneb offers numerous advantages, including low cost, strong activity, low acute toxicity, multiple action sites, and a low risk of resistance development. It is commonly used for fungal control in fields, plantations, nurseries, orchards, and forestry. Maneb targets fungi at diverse sites, inactivating them by interfering with sulfhydryl groups in amino acids or forming complexes with metal-containing enzymes, disrupting normal biochemical processes and metabolic balance in the fungal cytoplasm and mitochondria (4). Therefore, it is frequently combined with singlesite fungicides to help mitigate the development of fungal resistance (5). The widespread global use of maneb has led to increasing environmental pollution and health concerns. Environmental monitoring data show that maneb has been detected in environmental media worldwide, including surface water, drinking water, soil, and crops (6–8). Furthermore, toxicological studies have shown that maneb can cross the blood–brain barrier and accumulate in the central nervous system, leading to abnormal nerve conduction and damage to dopaminergic neurons, thus causing neurotoxicity (9, 10). However, research on the exposure status and toxic effects of maneb is currently incomplete. Most existing studies have concentrated on the combined toxicity and its mechanism of maneb with other pesticides, such as paraquat (PQ), while research on the toxicity of maneb’s itself remains incomplete. Moreover, the specific mechanism of maneb’s toxicity has not been fully elucidated, and the complete chain from its entry into the human body to the triggering of health damage is still unclear. Therefore, this research aims to summarize and explore the environmental residues, human exposure levels, and specific toxic effects of maneb, in order to provide a theoretical basis for the more rational application of maneb in agricultural and forestry fungal control. To establish the foundation for this review, extensive literature collection was conducted. The sources and scope of the literature included academic databases such as PubMed, Web of Science, Elsevier, etc. The time range focused on research conducted in the past two decades (2005–2025), and landmark early classic literature in this field was also included. The search keywords included “maneb,” “ETU,” “environmental residues,” “population exposure,” “neurodegenerative diseases,” “neurotoxicity,” etc. Literature screening was guided by the principles of theme relevance and scientific importance. Priority was given to including the following: (1) studies that solely explored the effect of maneb exposure; (2) studies with clear findings regarding the mechanism of neurotoxicity; (3) high - quality population epidemiological studies, *in vivo* animal experiments, and *in vitro* cell experiments. Based on this, the analysis and synthesis of the included literature are aimed at outlining the development trajectory, comparing different viewpoints, and identifying current knowledge gaps and future research directions.

## 2 Residual levels of maneb in the environment

Maneb readily decomposes upon exposure to air or moisture, with the resulting decomposition products largely influenced by environmental factors including soil composition, aqueous conditions, and light exposure. The decomposition products are shown in Table 1. The primary degradation product, ETU, has been shown to exhibit multiple teratogenic, carcinogenic, and immunotoxic properties, in addition to exhibiting some genotoxicity (11, 12). Maneb itself is insoluble in water, but ETU is stable at room temperature and pressure, decomposes slowly, and has a high solubility in water, making it susceptible to groundwater contamination (13). According to documents released by the United States Environmental Protection Agency (USEPA), the half-life of ETU in aerobic soil is approximately 3 days, the half-life in aerobic aquatic environments is approximately 6 days, and the half-life in anaerobic aquatic environments is as long as 149 days (14).

**Table 1:** Decomposition products of maneb.

Decomposition products	Source route	Environmental behavior
Dithiocarbamates	Hydrolysis intermediate	Convert to ETU
Mn^2+^	Metal ligands release	Adsorbed to soil
ETU	Main degradation products of hydrolysis, pyrolysis and microbial action	High water solubility, high mobility, high ecological risk, toxicity
CS_2_	Pyrolysis by-products	High volatility, toxicity
CO_2_、NH_3_	Fully mineralized product	Release to atmosphere

Environmental monitoring shows that maneb residues or degradation products can be detected in environmental media in areas with frequent maneb use (Table 2), with residues being more pronounced in areas of heavy or continuous application (7, 15). Long-term monitoring of maneb in the surface water of farmland irrigation areas in the central-eastern part of Ghana reached 120 μg/L (16). The average mancozeb-ETU concentration in drinking water in Paraná State, Brazil, was 121.4 μg/L, and ETU residues in 80.31% of municipalities were significantly above the pesticide use limit, directly or indirectly contributing to approximately 270 cancer cases (17). In a deep groundwater well in the United States, the concentration of ETU in the drinking water reached 700 μg/L (18).

**Table 2:** Residues of maneb in the environment.

Area	Substance	Medium	Concentration	Limiting value	References
Ghana	Maneb	Surface water	120 μg/L	48 μg/L	(16)
Brazil	Mancozeb-ETU	Drinking water	121.4 μg/L	0.1 μg/L	(17)
USA	ETU	Drinking water	700 μg/L	7 μg/L	(18)
Brazil	DTC	Strawberry	3.8 mg/kg (highest)	0.2 mg/kg	(20)
			0.46 mg/kg (average)		
		Banana	3.3 mg/kg (highest)	1 mg/kg	
		Tomato	3.3 mg/kg (highest)	2 mg/kg	
Yaoundé	Maneb + Mancozeb	Papaya	0.14 mg/kg	0.05 mg/kg	(22)
		Pineapple	0.10 mg/kg	0.5 mg/kg	
		Vanilla	8.66 mg/kg	5.0 mg/kg	
Spain	DTC	Lettuce	0.06–22.5 mg/kg	5 mg/kg	(23)
		Pepper	0.02–0.34 mg/kg	0.1 mg/kg	
Türkiye	Maneb	Tomato juice	0.45 mg/L	3 mg/kg	(24)
	ETU		0.08 mg/L	0.05 mg/kg	
			0.11 mg/L		
Europe (2019)	Maneb	Apple	333^1^	0.11 mg/kg	(25)
		Lettuce	239^1^		
		Peach	130^1^		
		Tomato	106^1^		
Europe (2020)	Maneb	Orange	145^1^	0.11 mg/kg	(26)
		Pear	403^1^		
Europe (2021)	Maneb	Melon	193^1^	0.11 mg/kg	(27)
		Table grapes	153^1^		
Europe (2022)	Maneb	Lettuce	166^1^	0.11 mg/kg	(28)
		Pear	252^1^		
		Table grapes	113^1^		

^1^The highest residual acute dietary risk assessment result (values are expressed as a percentage based on health-based acute reference value or ARfD; values > 100 indicate a certain health risk).

Among crops, the detection of dithiocarbamate pesticides (DTC) in samples of Danish fruits and vegetables, including maneb, revealed that 7.1% of the tested samples had DTC residues. The content of DTC in samples of spinach, cucumber, melon, apricot, pear, table grapes, sweet pepper, lettuce, podded legumes, and carrots was higher than the maximum residue limit (MRL) set by the European Union (19). In 520 crop samples from Brazil, the DTC residue rate reached 60.8%, with the average value of strawberry samples being 0.46 mg/kg and the highest content reaching 3.8 mg/kg, far exceeding the maximum residue limit of 0.2 mg/kg (20). The European Commission tested pesticide residues in botanical products from the EU, Norway, Iceland, and Liechtenstein, finding that maneb-type DTCs were most frequently detected and frequently exceeded the MRL (21). The total residue levels of maneb and mancozeb in papaya, pineapple, and vanilla from Yaoundé were 0.14, 0.10, and 8.66 mg/kg, all exceeding the previous MRL of 0.05, 0.05, and 5.0 mg/kg (22). In Spain, 93.33% of 150 fruit and vegetable samples, including apples, grapes, and lettuce, contained DTC pesticide residues, primarily EBDC pesticides such as maneb, mancozeb, and propineb. Six percent of the samples tested exceeded the MRL (23). In a tomato juice in a Turkish supermarket, the maneb residue content was 0.45 mg/L, while the other two tomato juices detected 0.08 mg/L and 0.11 mg/L of ETU residues (24). The monitoring results of the European Food Safety Authority (EFSA) in 2019 for pesticide residue levels in food on the European market indicated that the residue levels of maneb in apple, lettuce, peach, and tomato samples exceeded their respective acute concentration reference values (0.11 mg/kg bw) (25). The 2020 monitoring results indicated that maneb exceeded the residue limit in orange and pear samples (26). In 2021, maneb residue levels exceeded the recommended limit in melons and table grapes (27). In 2022, maneb exceeded the residue limit in lettuce, pear, and table grapes samples (28).

The residues of maneb and ETU in the environment enter the biological system through dietary intake, respiratory exposure, etc. Among them, since maneb is widely used for the sterilization and prevention of diseases in crops such as fruits and vegetables, the residues on food surfaces are one of the main sources of exposure for the population (29, 30). Long-term exposure to maneb residues may expose organisms to a range of health risks, including neurological damage, immune system suppression, and reproductive abnormalities (6, 31, 32). An environmental risk assessment of the aquatic and terrestrial environments in Ghana’s region, conducted applying the risk assessment model PRIMET (Pesticide Risks in the Tropics to Man, Environment and Trade) and the Species Sensitivity Distribution (SSD) method, indicated that maneb is one of the pesticides with the highest chronic toxicity risks for aquatic and terrestrial ecosystems in some areas of southern Ghana, and it also posed a certain acute risk to species such as bees (16). In conclusion, Chapter 2 summarizes the residual levels of maneb in the environment, which constitute potential sources of exposure for the population. It should be noted that the environmental residual concentration cannot be directly equated with the exposure dose or health risk in the human body. Quantitative estimation needs to be carried out through exposure pathways and exposure models (such as estimating daily intake, EDI).

## 3 Exposure level of maneb

### 3.1 General population exposure

The general population mainly obtains maneb and ETU through diet, mainly from vegetables, fruits and grains (33). After entering the body, maneb and its metabolites exist in organs such as the liver, kidneys, especially the thyroid gland, and are metabolized by hepatic microsomal enzymes to produce ETU. ETU is rapidly absorbed through the gastrointestinal tract, and its residues accumulate preferentially in the thyroid gland, followed by the liver (34, 35). Depending on their different characteristics, the measured residues of maneb in environmental media more directly indicate the initial pollution source, while the ETU detected in the human body, such as in urine, is a more reliable biomarker reflecting the actual internal exposure dose. Meanwhile, Mn is mainly accumulated in organs such as the liver and pancreas, with particularly high content in bones, and the brain is the main target organ for neurotoxicity (36, 37). According to the standards issued by the European Union in 2007, the acceptable daily intake (ADI) and acute reference dose (ARfD) for maneb are 50 μg·kg^−1^ and 200 μg·kg^−1^·day^−1^, used in chronic toxicity risk assessments and acute risk assessments (38).

The daily acute intake of maneb for adults and children in Denmark was 11.2 and 28.2 μg·kg^−1^·day^−1^, representing 5.6 and 14.1% of the adult ARfD (19). The acute and chronic dietary exposure of maneb for the US population was 14 and 0.081 μg·kg^−1^·day^−1^, with an ETU exposure concentration of 0.430 μg/mL and a cancer dietary risk of 9.6 × 10^−7^. The group with the highest exposure risk was American female citizens aged 13 to 49 years old, with acute and chronic dietary exposures of 18 and 0.103 μg·kg^−1^·day^−1^ (14, 39).

Studies have demonstrated that prolonged exposure to environments with excessive Mn can result in neurodegeneration, heightened inflammatory responses, and an elevated risk of developing neurodegenerative diseases (40). In 124 drinking water samples from households near a large banana plantation in Costa Rica, 94% of the samples tested positive for Mn residues, and 6% tested positive for ETU residues. Among these samples, 22 and 7% had Mn concentrations higher than 100 and 500 μg/L. The concentration of Mn in water samples was negatively correlated with the distance from the banana plantation (41). Among pregnant women, 72% had an excess daily intake of ETU above the reference dose, with the mean Mn concentrations in blood and hair samples of 24.4 μg/L and 1.8 μg/g (42, 43). Excessive prenatal exposure of pregnant women to Mn-based disinfectants can lead to impaired neural development and gender-specific effects in infants. High Mn concentrations result in lower cognitive levels in female infants and are associated with lower social emotional processing abilities in male infants (44). Another study also found that ETU concentrations were associated with neurobehavioral development in children aged 6–9 years in exposed areas. Children with higher urine ETU concentrations showed poorer language learning abilities. This study employed comprehensive and standardized neuropsychological tests, controlled for socioeconomic confounding factors, but the sample size was relatively small (*n* = 140). The cross-sectional design could not determine the causal relationship. It is not clear whether children with poor development or poor development due to exposure to maneb are more likely to exhibit high exposure behaviors (45). Additionally, exposure to Mn-based fungicides within 3 kilometers of residences in California resulted in elevated Mn concentrations in children’s dentin during prenatal life (46).

### 3.2 Occupational exposure

In addition to the general population, the occupational groups with direct or indirect exposed to maneb during related work processes such as production, transportation, storage, application and disposal face elevated exposure risks and an increased likelihood of adverse health effects. Existing studies have shown that occupational exposure to maneb can lead to neurological and hematopoietic system diseases as well as endocrine disorders (47). Common occupations include foliar spraying, seed treatment and soil treatment. The respiratory tract is the main way for maneb to enter the occupational population (48). To ensure credibility, this research focused on the research design, sample size, the accuracy of exposure assessment methods, and the control of key confounding factors such as age.

The risks caused by maneb occupational exposure to workers can be mainly classified into carcinogenic risks and non-carcinogenic risks. Non-carcinogenic risks are mainly evaluated through the margin of exposure (MOE) (49, 50). MOE is an assessment indicator for human and animal health risks, and is mainly used to evaluate the safety of substances in food or feed (51). In the assessment of the non-carcinogenic occupational risks of maneb, the MOE evaluation values for exposure routes such as mixing and transporting maneb, aerial spraying, and ground chemical irrigation were all less than 100, which indicates that there is a certain potential risk of maneb exposure in these processing procedures, underscoring that occupational risks are not negligible. In addition, the carcinogenic risk of the workers responsible for mixing and loading the wettable powder of maneb for aerial spraying was higher than the specified limit (1 × 10^−4^).

The occupational exposure risk of ETU, the main metabolite of maneb, is most significant among workers engaged in the production, preparation, and application of EBDC pesticides (52). Its average biological half-life is 7.9 h (53). In an Italian factory that produces Mn-containing EBDC pesticides, the detected concentrations of ETU in the air, hand-washing residues, and the body surface of the workers were 1.9 μg/m^3^, 36.9–194.3 μg, and 15–96 ng/m^2^. The highest ETU concentration was found in the urine of workers involved in the formulation process, reaching 55.4 μg/g creatinine (54). The concentration of ETU in the workers’ urine increased from 0.5–2.1 μg/L to 1.9–8.2 μg/L after exposure (55). During the application of EBDC pesticides, the concentration of ETU rose even more significantly (56, 57). Urinary ETU levels in women working in plantation agriculture and laundry in Costa Rica were 19 and 11% higher than those in the general population (43).

## 4 Neurotoxic effects and mechanisms of maneb

### 4.1 Neurotoxic effects of maneb

Environmental pollution caused by pesticide residues has become a global hot topic, and the presence of maneb in the environment is more serious than previously anticipated. Since 2017, maneb has been banned in countries such as the European Union, the United Kingdom, the United States, Australia, New Zealand, and Canada. However, it continues to be used in certain areas in developing countries such as Brazil, India, and China (58). Brazil accounts for 20% of global pesticide consumption, and in 2023, maneb usage ranked second among all pesticides used in Brazil (59). However, in most countries or regions, the regulatory oversight of maneb is becoming increasingly strict, and plans for its gradual phase-out are often under discussion or already in progress. Most toxic substances exposed to the environment are metabolized or degraded through various pathways. After crossing the blood–brain barrier, they accumulate in the central nervous system, disrupting neural development and affecting sensory, motor, and cognitive functions (60, 61). Moreover, in-depth research has revealed that maneb does have some toxicity to humans.

Epidemiological investigations have shown that the incidence of neurofunctional disorders among people with long-term exposure to maneb is significantly higher than that of the control group, with symptoms including abnormal motor coordination, cognitive decline, and sensory neuropathy, among others (62). Research indicates that combined exposure to maneb and paraquat triples the risk of neurodegenerative diseases in patients (63). It has been confirmed that it increases the expression of *β*-amyloid precursor protein (AβPP) and Aβ42 in dopaminergic nerve cells, and Aβ42 is closely related to the onset of Alzheimer’s disease (AD) (64, 65). Furthermore, two young agricultural workers developed Parkinson’s syndrome after long-term exposure to maneb. Further studies have shown that male farmers who were occupationally exposed to maneb had significantly higher rates of symptoms such as headache, fatigue, tension, memory loss, and sleepiness compared to the non-exposed group. Additionally, other neurological changes were also observed, such as postural tremors, cerebellar signs, and motor retardation (66).

Parkinson’s disease (PD) is a highly prevalent neurodegenerative disease. Its typical pathological features include the degeneration and loss of dopaminergic neurons in the substantia nigra pars compacta of the midbrain, and the formation of eosinophilic inclusions, Lewy bodies (LBs), in the remaining neurons. The main component of Lewy bodies is abnormally aggregated *α*-synuclein (α-syn) (67, 68). In our previous research, after 90 days of intraperitoneal injection of maneb at environmental dose (0.05 mg/kg bw) and high concentration (60 mg/kg bw), mice developed PD-like motor disorders, and abnormal aggregation of α-syn was observed in neurons. Similar neurotoxic were also observed in SH-SY5Y cells, PC12 cells, and neural stem cells (3, 32, 69, 70). The main active ingredient of maneb, manganese ethylenedithiocarbamate (Mn-EBDC), was shown to induce PD by inhibiting the activity of mitochondrial complex III. Its toxic effects were similar to those of the classic PD neurotoxin 1-methyl-4-phenylpyridine (MPP+) (71). Furthermore, maneb was also found to modify the activity of mitochondrial peroxiredoxin 3 (Prx3) through thiol modification, thereby affecting cellular energy metabolism and ultimately leading to the apoptosis of dopaminergic neurons and the occurrence of neurodegenerative disorders (72). Another study pointed out that after 6 weeks of continuous exposure to maneb, the damage to dopamine neurons in the substantia nigra pars compacta was caused by inducing hippocampal cognitive deficits and synaptic loss, and increasing the mRNA levels of microglial phagocytic marker CD68, immune adhesion factors ICAM1, ICAM2, and proinflammatory factors IL-6, IL-1β, CD11b, and TNF-*α* (73).

**Figure 2:**
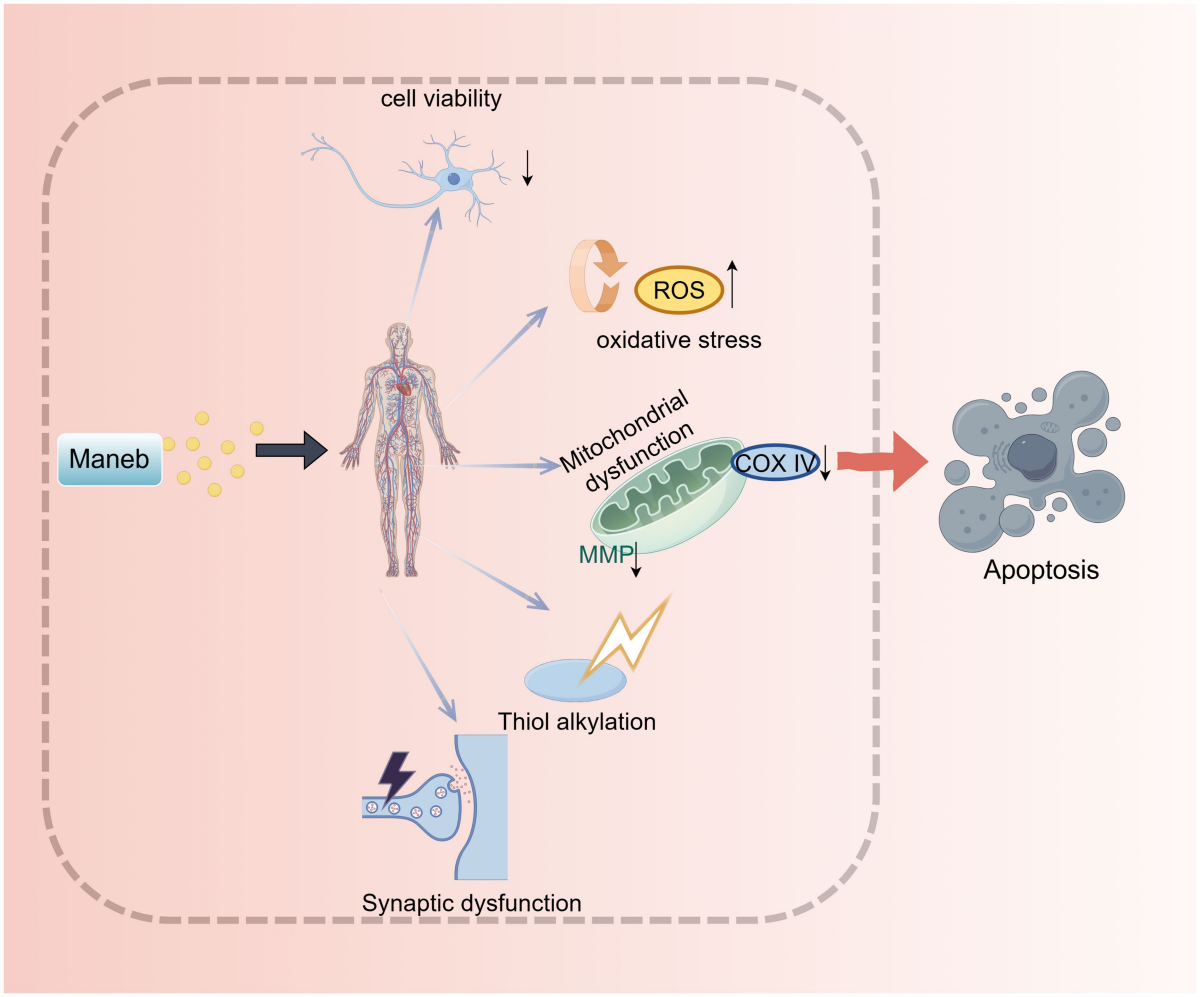
The neurotoxic effects of maneb.

This neurotoxic effect is closely related to the unique molecular mechanism of maneb. It not only can destroy the normal physiological structure of cells and interfere with the normal function of neurotransmitters, but also can induce neuronal damage through activating the oxidative stress pathway and damaging mitochondrial function, ultimately leading to cell apoptosis (Figure 2). These mechanisms work together to further exacerbate the damage to the central and peripheral nervous systems in the human body (31, 32, 74).

### 4.2 Neurotoxic mechanism of maneb

#### 4.2.1 Decreased cell viability

A large number of toxicological studies have demonstrated that maneb, whether exposed alone or in combination, can significantly reduce cell viability by disrupting cell membrane integrity and interfering with normal cellular energy metabolism. This effect is particularly pronounced on dopaminergic neurons. Our previous research has confirmed that after exposing dopaminergic neurons to maneb and its metabolite for 24 h, the cells showed signs of shrinkage, increased intercellular spaces, and reduced adhesion rates, with a decline in cell viability, suggesting a synergistic toxicity mechanism between the organic and metal ions in maneb (3). Similar cell viability inhibitory effects have also been observed in other studies. After acute exposure to maneb and mancozeb for 24 h, maneb-induced lipid peroxidation disrupted the cell membrane structure, and the viability of midbrain dopaminergic cells decreased. After 24 and 48 h of combined exposure to maneb and paraquat, SH-SY5Y cells viability was significantly decreased, cell membrane integrity was disrupted (75). In addition, maneb also has a cellular senescence-inducing effect. Cell cycle distribution analysis showed that SH-SY5Y cells were arrested in the S phase after maneb exposure, and the expression levels of senescence-related proteins such as p16, p38, ERK, and Rb were upregulated. The inhibitory effect on cell viability was time- and dose-dependent (76).

#### 4.2.2 Oxidative stress

Under normal physiological conditions, the use of oxygen by aerobic biological cells can produce potentially harmful reactive oxygen metabolites. These oxygen derivatives are collectively referred to as reactive oxygen species (ROS). An imbalance between prooxidants and antioxidants in the body can lead to excessive production of ROS within the body, causing oxidative stress. Oxidative stress may disrupt the normal structure and activity of proteins and further result in cell death (77). Among the cellular stressors, maneb can interfere with the normal metabolic mechanisms of cells through various redox reaction mechanisms, the most common of which is to increase the generation of ROS in different tissues and organs and cause oxidative stress (78–80).

Researches have found that maneb exposure increased the levels of ROS, 4-hydroxynonenal (4-HNE), and superoxide dismutase (SOD) activity in rat polymorphonuclear leukocytes (PMNs). PMNs experienced oxidative stress, and the expression levels of glutathione-S-transferase A4-4 (GSTA4-4) and inducible nitric oxide synthase (iNOS) also increased (81). Another research also found that exposure to maneb increased the levels of ROS, nitrotyrosine, lipid peroxidation (LPO), and nitrite in PMNs. Additionally, the catalytic activities of iNOS, SOD, and xanthine oxidase (XO) also increased (82). Similar phenomena were also observed in HepaRG cells, SH-SY5Y cells, and PC12 cells (3, 8, 75). Research have further demonstrated that maneb exposure produces ETU and Mn^2+^ through metabolism, leading to intracellular Mn^2+^ overload and Zn^2+^ depletion, which in turn induces oxidative stress and subsequently activates caspase-3 and caspase-9-dependent apoptosis (8). It is worth noting that the synergistic effect between Mn^2+^ and ETU is not simply additive. It may involve more complex toxicokinetic interactions. The hypothyroidism and systemic changes induced by ETU can increase the permeability of the blood–brain barrier, allowing Mn^2+^ to enter the brain more easily. At the same time, the combined effect of the overall slowdown of energy metabolism and functional impairment further exacerbates the neurotoxicity (83, 84). However, this viewpoint still requires future experimental studies to verify. Other researches have suggested that maneb activates the Nrf2/ARE pathway, upregulating glutamate cysteine ligase (GCL) and increasing the synthesis of reduced glutathione (GSH), ultimately leading to oxidative stress (75). Furthermore, similar findings were observed for the combined exposure of maneb and other toxic substances. For instance, the combined exposure of maneb and paraquat inhibited the proliferation of rat neural stem cells through oxidative stress, leading to abnormal expression of cell cycle regulatory factors such as cyclin D1, cyclin D2, Rb1, and p19, and inducing neurodevelopmental toxicity (85).

#### 4.2.3 Mitochondrial dysfunction

In neurons, mitochondria are crucial organelles for metabolism and Ca^2+^ homeostasis. From the development of damaged neurons to various neurodegenerative diseases, mitochondrial dysfunction and changes in mitochondrial dynamics are closely related (86–88). Researches have shown that oxidative stress induced by maneb also affects the cell death process through bcl-2 family proteins, activating Bax and Bak, further promoting the permeability of the outer mitochondrial membrane, thereby releasing death-inducing factors that lead to apoptosis and non-apoptotic death (68, 89).

Under subacute exposure, maneb was regarded as a redox regulator. It inhibited the normal function of mitochondrial complexes and the uncoupling of the mitochondrial proton gradient through protein thiol alkylation, inhibited ATP synthesis, significantly affected aerobic and anaerobic energy production, and ultimately led to cell apoptosis (90). Under acute high-dose exposure, maneb reduced ATP levels and impaired the normal energy metabolism function of cells through mitochondrial dysfunction and mitochondrial uncoupling, inhibited the activity of mitochondrial complex III, and further caused dose-dependent toxicity of dopamine (DA) and gamma-aminobutyric acid (GABA) in midbrain neurons of rat embryos (91). Similarly, combined exposure to maneb and paraquat also altered the expression of 14 proteins in the mitochondrial proteome of the substantia nigra striatum tissue, inducing mitochondrial dysfunction and leading to dopaminergic neurodegeneration in the substantia nigra striatum (92).

#### 4.2.4 Synaptic dysfunction

Synapses enable neurons to connect and communicate with each other, and are a prerequisite for the normal functioning of the brain. They play a crucial role in the nervous system, including the transmission, integration, and processing of information (93). Synaptic dysfunction is one of the earliest observed cellular defects in neurodegenerative diseases such as AD and PD (94).

Studies have shown that continuous exposure to maneb for 6 weeks will lead to hippocampal cognitive deficits and synaptic loss, and increase the mRNA levels of microglial phagocytosis-related CD68, ICAM1 and ICAM2, as well as pro-inflammatory factors IL-6, IL-1β, CD11b and TNF-*α*, ultimately inducing damage to dopamine neurons in the substantia nigra pars compacta (73). Another research has indicated that maneb and its metabolites have an oxidative stress-inducing effect on *Caenorhabditis elegans*, with concentration-dependent increases in glutathione and its oxidants. Furthermore, destabilization of neurotransmitters such as DA, acetylcholine, and GABA, as well as morphological changes in dopaminergic neurons were observed (31). Our previous research used A53T α-syn transgenic mice as the experimental mode through proteomics and metabolomics studies, also confirmed that maneb exposure disrupts protein and metabolite levels in neurotransmitter pathways, inducing PD-like neurotoxicity, including phenylalanine and tryptophan metabolic pathways, dopaminergic synapses, and synaptic vesicle cycling. Moreover, this induction effect can be inhibited by the asparagine endopeptidase (AEP) inhibitor Compound #11 (CP11) (95).

#### 4.2.5 Cell apoptosis

**Figure 3:**
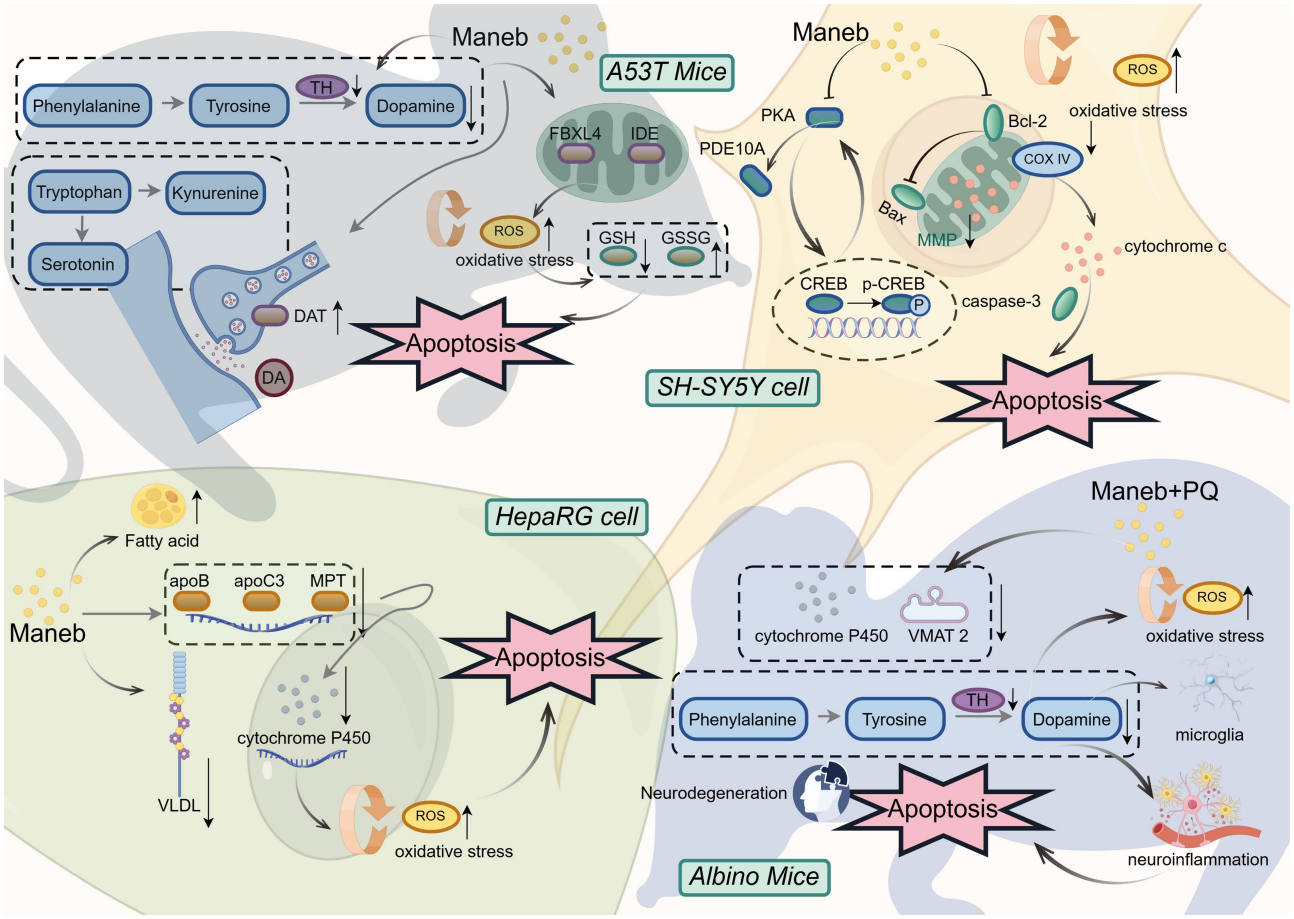
The mechanism of neurotoxic effects of maneb.

Apoptosis, also known as programmed cell death, involves a series of gene activation, expression and regulation. It is mostly mediated by various internal or external signaling pathways triggered by multiple factors (96, 97). Numerous studies have thoroughly elucidated the specific molecular mechanisms by which maneb exposure induces apoptosis (Figure 3), including disruption of intracellular Mn^2+^ and Zn^2+^ homeostasis, interference with lipid and xenobiotic metabolism, activation of Bcl-2 family proteins, and caspase-3/9-mediated intrinsic apoptotic pathways (8, 70, 98, 99).

Research indicated that maneb exposure alone induced Bak-dependent neuronal apoptosis by modulating the levels of the pro-apoptotic protein Bak. However, when co-exposed with paraquat, the two compounds acted synergistically to upregulate the expression of Bak inhibitors, thereby suppressing the Bak-mediated apoptotic pathway and shifting apoptosis induction to the Bax-dependent pathway (98). Another research using albino mice as an experimental model demonstrated that exposure to maneb and paraquat exacerbated oxidative stress and neuroinflammation by downregulating the expression of CYP2D22 and vesicular monoamine transporter 2 (VMAT-2), reducing the number of tyrosine hydroxylase (TH)-positive neurons, and decreasing dopamine levels, ultimately promoting neuronal apoptosis and neurodegeneration (100). Another research linked the activity of LRRK2 kinase to maneb-induced apoptosis. After 6 h of combined exposure to maneb and paraquat, the phosphorylation of LRRK2 kinase in human nerve-like cells significantly increased, caspase-3 was activated, leading to nuclear condensation and oxidative stress, and the mitochondrial membrane potential significantly decreased, resulting in cell apoptosis (79). Consistent with the pathological features of PD, exposure to maneb and paraquat triggered the release of cytochrome C from mitochondria into the cytoplasm, where its peroxidase activity promoted the formation and oligomerization of *α*-syn radicals, ultimately leading to neuronal apoptosis (101).

In addition, maneb exposure significantly altered the phospholipid and fatty acid composition in neurons and astrocytes, thereby disrupting cell membrane homeostasis. Neurons may release lipid ligands that activate FPR2/ALX receptors on glial cells, triggering the FPR2/ALX signaling pathway and exerting neuroprotective effects (102). Our research group demonstrated through *in vivo* and *in vitro* experiments that maneb reduced dopaminergic neuron viability in a concentration-dependent manner, impaired mitochondrial function, and mediated apoptosis through the mitochondrial apoptotic pathway and the PKA/CREB signaling pathway. Significant alterations were observed in the levels of key regulatory proteins, including Bcl-2, Bax, cytochrome c, PKA, PDE10A, CREB, and p-CREB. Furthermore, the PKA activator dbcAMP was shown to attenuate maneb-induced activation of the mitochondrial apoptotic pathway (32).

In addition to neurotoxicity, maneb exposure was confirmed to cause steatosis, interfere with the secretion of lipoproteins (VLDL), reduce the mRNA levels of apolipoprotein B, C3, and microsomal triglyceride transfer protein, and decrease the mRNA expression and activity levels of cytochrome P450, ultimately leading to apoptosis of HepaRG cells (99).

## 5 Conclusion

Since registrated in the early 1960s, maneb has been widely used in various countries and regions due to its high activity, low cost, diverse action sites, and low risk of developing resistance. It is regarded as one of the significant environmental risk factors and has triggered a series of chain reactions in terms of health. This article systematically presents the residual concentrations of maneb and its metabolites in environmental media, and separately summarizes the exposure levels of maneb for the general population and occupational populations. Subsequently, it analyzes the neurotoxic effects of maneb from the perspectives of effects and mechanisms. Epidemiological and *in vitro*/*in vivo* experimental results have confirmed that maneb exposure increases the probability of developing neurodegenerative diseases, especially PD, with abnormal aggregation and phosphorylation of *α*-syn, damage to dopaminergic neurons, and ultimately resulting in motor dysfunction. The specific mechanisms of its toxic damage include altering the normal physiological state of cells and reducing cell viability, increasing the level of ROS, inducing mitochondrial dysfunction, interfering with normal energy metabolism and neurotransmitter transmission in cells, and inducing cell apoptosis, etc. Future research can be conducted in the following key aspects in greater depth and detail: (1) Currently, most studies focus on the combined toxicity of Maneb with other pesticides. Therefore, it is necessary to clarify the specific toxicological characteristics of maneb when exposed alone. In addition, future research must also clarify the dose–response relationship of maneb, especially the *in vivo* toxicokinetic interactions and synergistic effects of its key metabolites ETU and Mn^2+^ under chronic and low-dose exposure conditions; (2) Future research should focus on studying the toxicity mechanism of maneb under more complex environmental stress conditions. By using multi-omics techniques, including transcriptomics, metabolomics and epigenomics, the interactions between maneb and other common agricultural chemicals, as well as environmental stress factors such as ultraviolet radiation and temperature changes, should be simulated to more accurately predict its comprehensive risk in agricultural environments; (3) To transform the toxic effects and mechanisms into risk management measures, the key lies in conducting application-oriented research, which includes: developing efficient remediation technologies for maneb and ETU contaminated sites, verifying exposure biomarkers for monitoring and compliance with regulatory requirements, such as ETU in urine, thereby converting the knowledge of mechanisms into practical solutions to mitigate the hazards caused by maneb exposure.
